# 

**Published:** 1918-12-28

**Authors:** 


**ORDER THE PAPER.**

**In consequence of the No Returns Order of the Government, readers of THE HOSPITAL are requested to ensure a regular supply of the paper by placing an order with their newsagent or bookstall without fail.**

### "Winter's Pie, 1918."

This cheery annual well maintains the reputation it has won for itself. It is full of good things, and if the suggestion of its Editor, Mrs. Hugh Spottiswoode, is followed, that all copies purchased by our readers might be subsequently sent on to hospitals, convalescent homes, and camps, the Navy or Army, we can prophesy that our wounded warriors will spend a merry hour over it. *Winter's Pie* is published at 6 Great New Street, London, E.C., and is obtainable at all booksellers' and bookstalls, 1s. 6d. net.

### CHRISTMAS ENTERTAINMENT.

Christmas at University College Hospital will be celebrated on a more festive scale this year than has been possible during the period of the War. An anonymous donor is providing a Christmas dinner for the soldiers under treatment in the Hospital, and the civil patients will be given special fare. The wards will be decorated and entertainments will be provided for all the patients by people interested in the Hospital. There will be a Christmas-tree for the children on Boxing Day. We hope the management will send us an account of all these merrymakings so that they may be fully described in our forthcoming issue.

